# Nutritional modulation of neuroinflammation in Alzheimer’s disease and mild cognitive impairment: a systematic review of clinical and preclinical evidence

**DOI:** 10.1186/s12877-026-08126-x

**Published:** 2026-08-25

**Authors:** Mahmoud AL Sayed, Ahmed Said, Mohamed Meselhy

**Affiliations:** 1 Department of Health Science Sports, Faculty of Sport Science, Benha University, Benha, Egypt; 2 Department of Sport Training and Kinesiology, Faculty of Sport Science, Benha University, Benha, Egypt; 3 Department of Theories and Application of Team Sports and Racket Sports, Faculty of Sport Science, Benha University, Benha, Egypt

**Keywords:** Neuroinflammation, Alzheimer's disease, Mild cognitive impairment, Omega-3 fatty acids, Ketogenic diet, Vitamin D, B-vitamins, Gut-brain axis, Immunometabolism

## Abstract

**Background:**

Neuroinflammationis a key contributor to the pathogenesis of Alzheimer’s disease (AD) and mild cognitive impairment (MCI). Nutritional interventions have been proposed as potential modulators of neuroinflammatory pathways; however, the consistency and strength of the available evidence remain uncertain. This systematic review evaluated the clinical and preclinical evidence regarding the effects of nutritional interventions on neuroinflammatory mechanisms relevant to AD and MCI.

**Methods:**

PubMed/MEDLINE, Embase, Scopus, and Web of Science were systematically searched for studies investigating nutritional interventions and neuroinflammatory outcomes. Eligible studies included randomized controlled trials and preclinical experimental investigations evaluating omega-3 fatty acids, ketogenic dietary interventions, B-vitamin supplementation, vitamin D, and microbiota-targeted strategies. Primary outcomes included inflammatory cytokines, neuroinflammatory signaling pathways, oxidative stress biomarkers, and glial activation. Methodological quality and risk of bias were assessed using study design-specific appraisal tools.

**Results:**

Twenty-two studies met the eligibility criteria, including ten clinical studies and twelve preclinical investigations. Overall, nutritional interventions were associated with modulation of neuroinflammatory biomarkers, including TNF-α, IL-1β, and IL-6, together with alterations in NF-κB and NLRP3 signaling, microglial activation, and oxidative stress pathways. The most consistent evidence supported omega-3 fatty acids and ketogenic dietary interventions, although evidence for ketogenic strategies was derived predominantly from preclinical studies. Evidence for B-vitamin supplementation, vitamin D, and microbiota-targeted interventions was limited because relatively few eligible studies were available. Considerable heterogeneity was observed across intervention protocols, outcome measures, and study populations.

**Conclusions:**

Nutritional interventions may modulate biological pathways involved in neuroinflammation; however, the strength and certainty of the evidence vary across intervention categories. Larger, well-designed randomized controlled trials incorporating standardized neuroinflammatory biomarkers are required to clarify the therapeutic potential of nutritional strategies for AD and MCI.

**Supplementary Information:**

The online version contains supplementary material available at 10.1186/s12877-026-08126-x.

## Introduction

 Neuroinflammation is increasingly recognized as a central pathological process in Alzheimer’s disease (AD) and mild cognitive impairment (MCI), contributing to both disease onset and progression. Under physiological conditions, immune responses within the central nervous system (CNS) are tightly regulated and play essential roles in maintaining neuronal homeostasis and protecting the brain from injury and infection. In contrast, persistent activation of microglia and astrocytes promotes sustained production of pro-inflammatory mediators, including tumor necrosis factor-alpha (TNF-α), interleukin (IL)-1β, IL-6, reactive oxygen species (ROS), and nitric oxide. This chronic inflammatory environment contributes to synaptic dysfunction, neuronal injury, and cognitive decline [1, 2]. Neuroinflammation is now recognized as an integral component of AD pathophysiology that interacts closely with amyloid-beta (Aβ) accumulation, tau hyperphosphorylation, mitochondrial dysfunction, and progressive neurodegeneration. Activated microglia and astrocytes not only produce inflammatory mediators but also regulate amyloid clearance, tau propagation, synaptic function, and neuronal homeostasis, thereby establishing a self-perpetuating cycle that accelerates disease progression [3, 4].

Growing interest has focused on modifiable lifestyle factors that may influence neuroinflammatory processes, with nutrition emerging as an important determinant of brain health. Dietary components may regulate neuroimmune signaling through multiple mechanisms, including modulation of microglial activation, regulation of nuclear factor kappa B (NF-κB) signaling, attenuation of oxidative stress, and interactions with the gut-brain axis, thereby supporting the rationale for nutritional interventions targeting neuroinflammatory pathways in AD and MCI [5, 6].

A broad range of nutritional interventions has been investigated for their potential to modulate neuroinflammation, including dietary fatty acids, ketogenic dietary strategies, vitamins, probiotics, polyphenols, antioxidants, and other bioactive compounds [5–10]. In the present systematic review, nutritional interventions were not predefined before the literature search. Instead, a comprehensive systematic search was conducted to identify all eligible nutritional approaches investigated in AD or MCI that fulfilled the predefined eligibility criteria. The intervention categories synthesized in this review, namely omega-3 fatty acids, ketogenic dietary interventions, B-vitamin supplementation, vitamin D, and microbiota-targeted strategies, emerged from the available evidence that satisfied the inclusion criteria and reported neuroinflammatory outcomes relevant to the objectives of this review. Other nutritional interventions identified during the screening process, including polyphenolic compounds and antioxidant-based interventions, were excluded from the final synthesis because they did not satisfy the predefined eligibility criteria or did not report neuroinflammatory outcomes relevant to the objectives of this review.

Among the nutritional interventions identified through the systematic search, omega-3 polyunsaturated fatty acids (PUFAs), particularly eicosapentaenoic acid (EPA) and docosahexaenoic acid (DHA), have received considerable attention because of their anti-inflammatory properties. These fatty acids serve as precursors of specialized pro-resolving mediators, including resolvins and protectins, which actively promote the resolution of inflammation and restoration of tissue homeostasis [11, 12]. Ketogenic dietary interventions have also been investigated for their capacity to modulate neuroinflammatory pathways through effects on immunometabolism and mitochondrial function. In particular, β-hydroxybutyrate has been reported to regulate inflammatory signaling through inhibition of the NLRP3 inflammasome and modulation of mitochondrial energy metabolism [13, 14]. B-vitamin supplementation, vitamin D, and microbiota-targeted interventions have also been investigated for their potential to influence neuroinflammatory pathways through modulation of homocysteine metabolism, immune regulation, and gut-brain communication [15–18].

Because the objective of the present review was to evaluate the potential of nutritional interventions to modulate neuroinflammation, the synthesis deliberately focused on inflammatory and oxidative mechanisms while acknowledging their close interaction with the principal pathological features of AD and MCI. Despite growing interest in nutritional modulation of neuroinflammation, the available evidence has not been comprehensively synthesized with a specific focus on AD and MCI while integrating both clinical and preclinical evidence. Furthermore, the certainty of evidence varies substantially across nutritional intervention categories, and the relationship between mechanistic findings and clinical outcomes remains incompletely understood. Therefore, this systematic review aimed to synthesize the available clinical and preclinical evidence evaluating nutritional interventions that fulfilled the predefined eligibility criteria and investigated neuroinflammatory mechanisms relevant to AD and MCI. By integrating mechanistic findings from experimental studies with evidence from human investigations, this review sought to evaluate the potential role of nutritional interventions in modulating neuroinflammation while critically considering the methodological quality, certainty, and limitations of the available evidence.

## Materials and methods

### Protocol and registration

This systematic review was conducted in accordance with the Preferred Reporting Items for Systematic Reviews and Meta-Analyses (PRISMA) 2020 Statement [7]. The review methodology was developed following the recommendations of the Cochrane Handbook for Systematic Reviews of Interventions [8]. The review protocol was prospectively registered with the International Prospective Register of Systematic Reviews (PROSPERO) (registration number: CRD420261345821). The completed PRISMA 2020 checklist is provided in Supplementary File S1.

### Eligibility criteria

Study eligibility was established using the Participants, Intervention, Comparators, Outcomes, and Study design (PICOS) framework. This approach ensured transparent and systematic identification of studies relevant to the objectives of the review. The predefined eligibility criteria are summarized in Table 1.

**Table 1:** Inclusion and exclusion criteria

Category	Inclusion Criteria	Exclusion Criteria
Population	Human participants diagnosed with Alzheimer’s disease (AD) or mild cognitive impairment (MCI); and experimentally validated animal or in vitro models investigating neuroinflammatory, metabolic, mitochondrial, or neuroimmune mechanisms relevant to AD/MCI-associated pathological pathways.	Clinical studies involving neurological disorders other than AD/MCI, including Parkinson’s disease, multiple sclerosis, stroke, traumatic brain injury, or other non-Alzheimer neurodegenerative diseases; and preclinical studies whose findings were not relevant to the biological pathways investigated in relation to AD/MCI
Intervention	Dietary nutrients (e.g., omega-3 fatty acids, vitamins, polyphenols, amino acids), nutrient supplementation, defined dietary patterns (e.g., ketogenic diet, Mediterranean diet), probiotics, prebiotics, dietary fiber, or gut microbiota–modulating nutritional strategies	Combined lifestyle interventions (e.g., diet plus exercise) where the isolated effect of the nutritional component could not be determined
Comparator	Placebo, standard care, untreated controls, or alternative dietary regimens; control groups in experimental models	Studies without comparator groups (except mechanistic in vitro studies with relevant neuroinflammatory outcomes)
Outcomes	Studies reporting at least one neuroinflammatory outcome relevant to the biological pathways implicated in Alzheimer’s disease (AD) or mild cognitive impairment (MCI). Eligible outcomes included neuroinflammatory biomarkers (e.g., TNF-α, IL-1β, IL-6), markers of microglial or astrocytic activation, neuroinflammatory signaling pathways (e.g., NF-κB and NLRP3 inflammasome), oxidative stress-related neuroinflammatory biomarkers, neuroimaging indicators of neuroinflammation, or histopathological evidence of neuroinflammatory changes.	Studies reporting only clinical, cognitive, or neurological outcomes without assessment of inflammatory biomarkers, neuroinflammatory mechanisms, or related biological pathways relevant to the objectives of the review
Study Design	Randomized controlled trials (RCTs), non-randomized trials, cohort studies, case-control studies, experimental animal studies, in vitro mechanistic studies	Case reports, narrative reviews, editorials, letters, conference abstracts without full data
Language	English	Non-English publications
Publication Date	1 January 2000–28 February 2026	Published before 2000
Publication Type	Peer-reviewed full-text articles	Grey literature without sufficient methodological detail

#### Participants

Eligible clinical studies included human participants diagnosed with Alzheimer’s disease (AD) or mild cognitive impairment (MCI). Preclinical evidence included experimentally validated animal or in vitro models used to investigate neuroinflammatory, metabolic, mitochondrial, or neuroimmune mechanisms relevant to pathways implicated in AD/MCI. Clinical studies involving other neurological disorders, including Parkinson’s disease, multiple sclerosis, stroke, traumatic brain injury, or other non-Alzheimer neurodegenerative diseases, were excluded. Preclinical models were considered only when their findings provided mechanistic evidence directly relevant to the biological pathways examined in this review and were interpreted as supportive mechanistic evidence rather than direct evidence of clinical efficacy in AD or MCI.

#### Interventions

No restrictions were imposed on the type of nutritional intervention during the literature search. Eligible interventions included specific nutrients (e.g., omega-3 fatty acids, vitamins, polyphenols, amino acids), dietary supplementation, defined dietary patterns (e.g., ketogenic diet and Mediterranean diet), and nutritional approaches targeting the gut microbiota (e.g., probiotics, prebiotics, and dietary fiber).

Studies evaluating combined lifestyle interventions, such as diet combined with exercise, were eligible only when the independent effects of the nutritional intervention could be clearly distinguished. Following systematic screening, studies were included in the qualitative synthesis only if they satisfied all predefined eligibility criteria and evaluated nutritional interventions relevant to AD/MCI-associated neuroinflammatory or related biological pathways while reporting eligible neuroinflammatory outcomes.

#### Comparators

Eligible comparator groups included placebo, standard care, alternative dietary or supplementation regimens, and untreated experimental controls for preclinical studies. Studies without comparator groups were excluded unless they provided mechanistic in vitro evidence directly relevant to neuroinflammatory pathways.

#### Outcomes

Eligible studies were required to report at least one outcome related to neuroinflammation within the central nervous system or a biological pathway directly relevant to neuroinflammatory mechanisms implicated in AD/MCI. Acceptable outcomes included inflammatory cytokines, such as TNF-α, IL-1β, and IL-6; markers of microglial or astrocytic activation; activation of the nuclear factor kappa B (NF-κB) signaling pathway; inflammasome-related signaling; oxidative stress biomarkers associated with neuroinflammation; C-reactive protein (CRP) when interpreted within the context of neuroinflammatory mechanisms; neuroinflammatory neuroimaging biomarkers; and histopathological evidence of neuroinflammation.

Studies reporting only clinical or cognitive outcomes without assessment of neuroinflammatory biomarkers or inflammatory mechanisms were excluded. Although amyloid-beta (Aβ) pathology, tau pathology, synaptic dysfunction, and neuronal degeneration represent key pathological features of AD, studies evaluating these outcomes alone were not eligible unless neuroinflammatory biomarkers or inflammatory mechanisms were also assessed. This approach ensured that study selection remained consistent with the predefined objectives of the review while acknowledging the close interaction between neuroinflammation and other pathological processes in AD.

#### Study design

Eligible study designs included randomized controlled trials, non-randomized controlled trials, cohort studies, case-control studies, experimental animal studies, and mechanistic in vitro investigations. Case reports, narrative reviews, systematic reviews, editorials, commentaries, letters, conference abstracts without full-text availability, and other non-original publications were excluded.

Only peer-reviewed articles published in English between 1 January 2000 and 28 February 2026 were considered eligible.

### Information sources and search strategy

A comprehensive literature search was conducted to identify studies published between 1 January 2000 and 28 February 2026. Four electronic databases were systematically searched: PubMed/MEDLINE, Embase, Scopus, and Web of Science Core Collection.

To maximize retrieval of eligible studies, additional search strategies were employed. The reference lists of all included full-text articles and relevant review articles were manually screened to identify potentially eligible publications. Forward citation tracking was performed using Scopus and Web of Science. Grey literature was searched through Google Scholar, with the first 200 records sorted by relevance being screened. Ongoing or unpublished clinical trials were identified through ClinicalTrials.gov. All retrieved records were imported into reference management software, and duplicate records were removed using automated detection followed by manual verification before study screening.

The search strategy was developed around three predefined conceptual domains: (1) neuroinflammation and central nervous system inflammatory processes, (2) nutritional interventions and dietary components, and (3) Alzheimer’s disease and related cognitive disorders. Both controlled vocabulary terms, including Medical Subject Headings (MeSH) in PubMed, and free-text keywords were used. Boolean operators (AND, OR), truncation symbols (*), phrase searching, and database-specific field tags were applied as appropriate for each database.

A representative PubMed search strategy combined terms related to neuroinflammation (e.g., *“neuroinflammation*,*” “microglial activation*,*”* and *“astrocytes”*), nutritional interventions (e.g., *“omega-3 fatty acids*,*” “vitamin D*,*” “polyphenols*,*” “ketogenic diet*,*”* and *“probiotics”*), and neurological disorders (e.g., *“Alzheimer disease*,*” “Parkinson disease*,*” “multiple sclerosis*,*” “stroke*,*”* and *“traumatic brain injury”*). Although search terms initially encompassed several neurological disorders to maximize search sensitivity, only studies involving Alzheimer’s disease or mild cognitive impairment that satisfied the predefined eligibility criteria were included in the final review. The complete search strategies for all databases are provided in Supplementary Table S2 to ensure transparency and reproducibility.

### Study selection

All records identified through electronic database searches and supplementary sources were imported into reference management software for screening. Duplicate records were removed using automated detection, followed by manual verification to ensure accuracy before study selection.

Study selection was conducted in accordance with the PRISMA 2020 guidelines using a two-stage screening process performed independently by two reviewers (M.AL.S. and M.M.).

#### Stage 1: Title and abstract screening

Titles and abstracts of all retrieved records were independently screened against the predefined eligibility criteria. Studies that clearly failed to meet the inclusion criteria were excluded at this stage. The principal reasons for exclusion included:

- Irrelevance to neuroinflammation.
- Absence of a nutritional intervention or dietary exposure.
- Non-original publications (e.g., reviews, editorials, commentaries, or conference abstracts).
- Clearly unrelated disease populations.

Disagreements between reviewers were resolved through discussion. When consensus could not be reached, a third senior reviewer adjudicated the final decision.

#### Stage 2: Full-text assessment

Full-text articles considered potentially eligible were retrieved and independently assessed by the same two reviewers using the complete predefined eligibility criteria. Each study was evaluated according to:

- Study design.
- Type of nutritional intervention.
- Presence of eligible neuroinflammatory outcomes.
- Appropriateness of the comparator group.
- Adequacy of methodological reporting.

Reasons for exclusion at the full-text stage were documented and are presented in the PRISMA 2020 flow diagram to ensure transparency.

Several studies investigating nutritional interventions in neurological disorders other than AD or MCI, including Parkinson’s disease, multiple sclerosis, stroke, and traumatic brain injury, were identified during screening. Although these studies were retrieved because of the broad search strategy, they were excluded during full-text assessment because they did not satisfy the predefined eligibility criteria restricting the review to AD and MCI.

The most common reasons for full-text exclusion were:

- Absence of eligible neuroinflammatory outcome measures.
- Combined lifestyle interventions in which the independent effect of nutrition could not be determined.
- Inadequate methodological design.
- Insufficient outcome reporting.
- Study populations outside the predefined scope of the review.

Inter-reviewer agreement during full-text assessment was evaluated using Cohen’s kappa coefficient, demonstrating substantial agreement (κ = 0.82).

Studies meeting all eligibility criteria were included in the qualitative synthesis. Because substantial heterogeneity was observed across study populations, nutritional interventions, outcome measures, and experimental methodologies, quantitative meta-analysis was not considered appropriate.

Following application of the predefined eligibility criteria, the included studies were categorized into five principal nutritional intervention groups:

1. Omega-3 fatty acids.
2. Ketogenic dietary interventions.
3. B-vitamin supplementation.
4. Vitamin D supplementation.
5. Microbiota-targeted interventions.

These categories emerged from the systematic screening process and formed the framework for the qualitative synthesis.

### Data extraction

Data extraction was performed independently by two reviewers (M.AL.S. and M.M.) using a standardized, pilot-tested data extraction form developed specifically for this review. Before formal data extraction, the form was piloted using five randomly selected eligible studies to ensure consistency, clarity, and uniform interpretation of the extracted variables. Minor revisions were made to improve completeness and reproducibility.

The following information was extracted from each included study:

#### Study characteristics

- First author and year of publication.
- Country of origin.
- Study design.
- Sample size.
- Participant or experimental model characteristics, including age, sex, and health status where applicable.

#### Intervention characteristics

- Type of nutritional intervention (e.g., omega-3 fatty acids, ketogenic dietary interventions, B-vitamin supplementation, vitamin D, probiotics, polyphenols, or other dietary approaches).
- Dosage and formulation, when reported.
- Duration of the intervention.
- Comparator or control condition.

#### Outcome measures

The primary outcomes extracted were neuroinflammatory biomarkers and related mechanistic measures, including:

- Inflammatory cytokines (e.g., TNF-α, IL-1β, IL-6, and CRP).
- Markers of microglial and astrocytic activation.
- NF-κB and other inflammatory signaling pathways.
- Oxidative stress biomarkers.
- Neuroinflammatory neuroimaging measures, when available.

#### Study findings

The following information was extracted for each study:

- Direction and magnitude of the intervention effect on neuroinflammatory outcomes.
- Statistical significance of the reported findings.
- Adverse events, where available.

For randomized controlled trials, additional methodological information was collected, including randomization procedures, allocation concealment, blinding, participant attrition, and funding sources.

All extracted data were independently cross-checked by both reviewers to ensure accuracy and completeness. Any discrepancies were resolved through discussion and consensus. When essential information was missing or unclear, attempts were made to contact the corresponding authors for clarification.

Because substantial heterogeneity was observed across study designs, participant populations, nutritional interventions, intervention duration, biomarker panels, and outcome assessment methods, the findings were synthesized qualitatively rather than quantitatively.

### Methodological quality assessment

The methodological quality of the included studies was assessed according to study design using validated risk-of-bias assessment tools.

The methodological quality of the clinical randomized controlled trials (*n* = 10) was evaluated using the Cochrane Risk of Bias 2 (RoB 2) tool [9]. This instrument assesses five domains of potential bias: (1) bias arising from the randomization process, (2) bias due to deviations from intended interventions, (3) bias due to missing outcome data, (4) bias in outcome measurement, and (5) bias in the selection of the reported result. Each study was assigned an overall judgment of low risk of bias, some concerns, or high risk of bias, according to the RoB 2 guidance.

The methodological quality of the animal studies (*n* = 10) was assessed using the SYRCLE Risk of Bias tool (Systematic Review Centre for Laboratory Animal Experimentation), an adaptation of the Cochrane risk-of-bias framework for animal intervention studies [10]. The SYRCLE tool evaluates ten domains, including sequence generation, baseline characteristics, allocation concealment, random housing, blinding of caregivers and investigators, random outcome assessment, blinding of outcome assessment, incomplete outcome data, selective outcome reporting, and other potential sources of bias.

Because no validated risk-of-bias instrument is currently available for mechanistic in vitro studies, the two in vitro investigations (*n* = 2) were evaluated qualitatively according to key indicators of methodological rigor, including the use of appropriate control conditions, experimental replication, transparency of intervention protocols, validity of outcome measurements, and completeness of reporting.

Quality assessment was conducted independently by two reviewers (M.AL.S. and M.M.). Any disagreements were resolved through discussion until consensus was achieved. When necessary, a third reviewer was consulted to resolve unresolved discrepancies. The results of the methodological quality assessment are presented in Tables 3, 4 and 5.

### Synthesis of results

A qualitative synthesis was undertaken because of substantial heterogeneity across the included studies with respect to study design, participant characteristics, experimental models, nutritional interventions, intervention duration, biomarker assessment, and outcome reporting. Clinical and preclinical evidence were synthesized separately to preserve methodological consistency and facilitate translational interpretation.

Clinical studies were grouped according to nutritional intervention category, including omega-3 fatty acids, ketogenic dietary interventions, B-vitamin supplementation, vitamin D, microbiota-targeted interventions, and other eligible nutritional strategies. The synthesis focused on predefined neuroinflammatory outcomes, including circulating inflammatory cytokines (e.g., TNF-α, IL-1β, and IL-6), C-reactive protein (CRP), oxidative stress biomarkers, and gut-brain axis-related inflammatory markers. For each intervention category, the direction of effect, statistical significance, and consistency of findings were evaluated.

Preclinical studies were synthesized according to their principal mechanistic targets, including microglial activation, astrocytic responses, NF-κB signaling, NLRP3 inflammasome activation, oxidative stress, mitochondrial function, and gut-brain axis modulation. Particular emphasis was placed on identifying biological pathways consistently influenced across experimental models and evaluating their potential translational relevance to Alzheimer’s disease and mild cognitive impairment.

A quantitative meta-analysis was not performed because substantial methodological heterogeneity precluded meaningful statistical pooling. Variability in study populations, experimental models, nutritional interventions, intervention duration, biomarker panels, and outcome assessment methods limited direct comparison across studies. Consequently, findings were synthesized narratively and summarized in tables to facilitate comparison between intervention categories and study designs.

Because the included evidence comprised randomized controlled trials, animal experiments, and mechanistic in vitro investigations, a formal GRADE assessment was not considered appropriate. Instead, the overall certainty of evidence for each nutritional intervention category was evaluated using a structured narrative approach based on four predefined domains: methodological quality, consistency of findings, directness of the available evidence, and translational relevance. This approach provided a balanced interpretation of the available evidence while acknowledging differences in study design and evidence hierarchy.

## Result

To facilitate interpretation of the findings, the included evidence is presented according to study design. Clinical studies evaluating nutritional interventions in individuals with Alzheimer’s disease (AD) or mild cognitive impairment (MCI) are summarized separately from preclinical animal and mechanistic in vitro studies. This structure reflects the hierarchy of evidence and distinguishes observations directly applicable to patient populations from mechanistic findings derived from experimental models.

### Search results

The systematic literature search identified 1,990 records through electronic database searching, comprising 468 records from PubMed/MEDLINE, 612 from Embase, 529 from Scopus, and 381 from Web of Science. After removal of 742 duplicate records, 1,248 unique citations remained for title and abstract screening.

Following initial screening, 1,110 records were excluded because they did not meet the predefined eligibility criteria. The most common reasons for exclusion were the absence of a nutritional intervention, failure to evaluate neuroinflammatory outcomes, non-original publications (e.g., reviews, editorials, or conference abstracts), exclusively in vitro studies without translational relevance, and study populations unrelated to Alzheimer’s disease (AD) or mild cognitive impairment (MCI).

A total of 138 full-text articles were assessed for eligibility. Of these, 116 studies were excluded for one or more of the following reasons: absence of eligible neuroinflammatory outcome measures (*n* = 39), observational study design without a nutritional intervention (*n* = 24), exclusively in vitro methodology (*n* = 18), insufficient methodological or statistical reporting (*n* = 17), ineligible study population (*n* = 11), or publication in languages other than English (*n* = 7).

Several studies investigating nutritional interventions in neurological disorders other than AD or MCI, including Parkinson’s disease, multiple sclerosis, stroke, and traumatic brain injury, were identified during full-text assessment. These studies were excluded because they did not satisfy the predefined eligibility criteria restricting the review to AD and MCI.

**Fig. 1:**
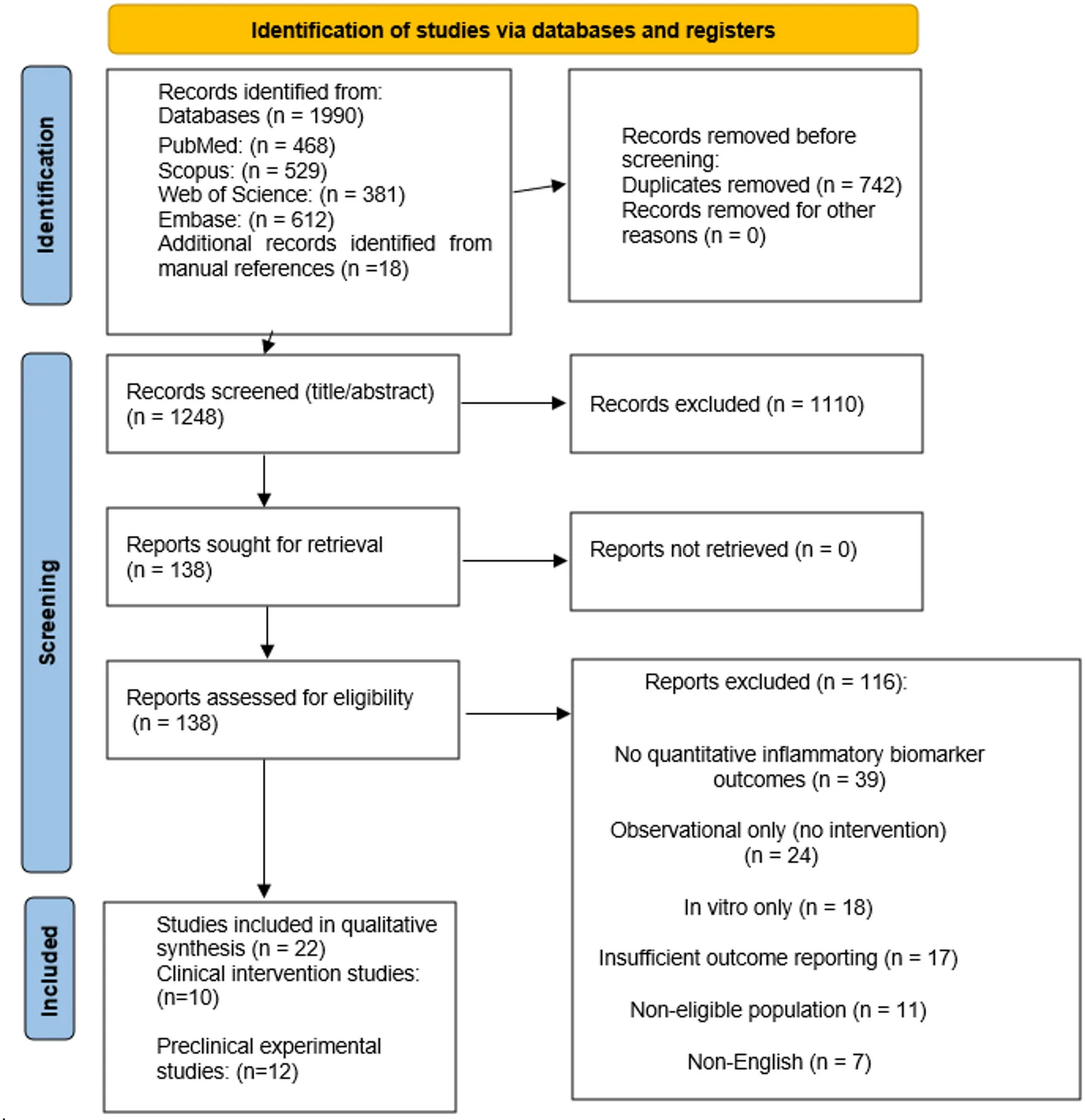
PRISMA flow diagram of study selection

Ultimately, 22 studies fulfilled all eligibility criteria and were included in the qualitative synthesis. These comprised 10 clinical intervention studies and 12 preclinical investigations, including 10 animal studies and 2 mechanistic in vitro studies. The complete study selection process is presented in the PRISMA 2020 flow diagram (Fig. 1).

### Study characteristics

The characteristics of the included studies are summarized in Table 2. A total of 22 studies met the eligibility criteria, comprising 10 clinical intervention studies and 12 preclinical investigations. The clinical evidence consisted of nine randomized controlled trials and one pilot randomized trial, whereas the preclinical evidence included 10 animal experiments and 2 mechanistic in vitro studies.

**Table 2:** Characteristics of included clinical and preclinical studies investigating nutritional modulation of neuroinflammation (*n* = 22)

**A.** Clinical Studies (*n* = 10)								
First Author (Year)	Country	Study Design	Sample Size	Population Characteristics	Intervention (Dose; Duration)	Comparator	Neuroinflammatory Outcomes	Key Findings
Lin (2022) [19]	Taiwan	Randomized, placebo-controlled	57	AD & MCI; elderly	Omega-3 (2 g/day DHA + EPA); 6 mo	Placebo	IL-6, TNF-α, CRP	↓ IL-6 (*p* < 0.05); TNF-α trend ↓; safe
Nagpal (2019) [20]	USA	Randomized crossover	17	MCI; older adults	Modified Mediterranean-KD; 6 wk	AHA diet	SCFAs; inflammatory metabolites	Improved anti-inflammatory SCFA profile
Nagpal (2020) [21]	USA	Randomized crossover	17	MCI; older adults	MMKD; 6 wk	Control diet	Gut mycobiome; inflammatory metabolites	Anti-inflammatory microbiome shift
Ma (2019) [22]	China	Single-blind RCT	180	MCI; elderly	Folic acid 400 µg ± B12; 6 mo	Control	IL-6, TNF-α	Significant ↓ IL-6 & TNF-α
Ma (2019) [23]	China	RCT	168	MCI; elderly	Folic acid 400 µg; 12 mo	Placebo	Aβ40/42; inflammatory markers	↓ Aβ-related neuroinflammatory biomarkers
Kwok (2020) [24]	Hong Kong	Placebo-controlled RCT	279	MCI; mean age > 70	B-vitamin complex; 24 mo	Placebo	CRP	Modest ↓ CRP; not robustly significant
Yang (2020) [25]	China	12-month RCT	183	MCI; elderly	Vitamin D 800 IU/day; 12 mo	Placebo	Oxidative stress markers	↓ oxidative stress (*p* < 0.01)
Stavrinou (2020) [26]	Cyprus	RCT	67	MCI; older adults	Omega-3/6 + antioxidants; 6 mo	Placebo	IL-6; oxidative stress	Significant ↓ IL-6 & oxidative stress
Shinto (2014) [27]	USA	Pilot RCT	39	Mild–moderate AD	Omega-3 ± alpha-lipoic acid; 12 mo	Placebo	TNF-α; IL-6	Downward inflammatory trend
Freund-Levi (2006) [28]	Sweden	Double-blind RCT	174	Mild–moderate AD	Omega-3 (1.7 g DHA + 0.6 g EPA); 6 mo	Placebo	IL-6 (subgroup)	↓ IL-6 in mild AD subgroup

B. Preclinical Studies (*n* = 12)								
First Author (Year)	Model	Study Type	Sample Size	Intervention	Duration	Neuroinflammatory Outcomes	Key Findings	
Xu (2022) [29]	AD mice	Animal experiment	40	Ketogenic diet	3 Mo	TNF-α; IL-1β; microglial activation	↓ cytokines; ↓ microglial activation	
Dilimulati (2023) [30]	rmTBI mice	Animal experiment	36	Ketogenic diet	8 wk	IL-1β; NF-κB	↓ NF-κB signaling	
Reilly (2020) [31]	APP/PS1 mice	Animal experiment	30	High-fat diet	5 Mo	TNF-α; IL-6	↑ neuroinflammation	
Aso (2013) [32]	APP/PS1 mice	Animal experiment	24	KD + triheptanoin	5 Mo	Cytokines; mitochondrial markers	↓ inflammatory signaling	
Beckett(2013) [33]	AD mice	Animal experiment	21	Ketogenic diet	6 wk	Inflammatory markers	Minimal change	
Brownlow (2013) [34]	AD mice	Animal experiment	32	Ketogenic diet	16 wk	Microglial activation	↓ activated microglia	
Hernandez (2018) [35]	Aged rats	Animal experiment	28	Ketogenic diet	12 wk	IL-1β; astrocyte markers	↓ IL-1β; improved synaptic markers	
Kashiwaya (2013) [36]	AD model	Experimental	30	Ketone ester	3 mo	NF-κB pathway	↓ NF-κB activation	
Ma (2018) [37]	Healthy mice	Animal experiment	24	Ketogenic diet	8 wk	Neurovascular inflammatory markers	Improved vascular inflammatory profile	
Ou (2020) [38]	AD mice	Animal experiment	32	Akkermansia muciniphila	6 wk	IL-1β; microglial markers	↓ microglial activation	
Sabokdast (2015) [39]	Microglial cells	In vitro	—	β-hydroxybutyrate	48 h	Oxidative stress; apoptosis	↓ oxidative stress & apoptosis	
Hu (2017) [40]	AD cellular model	In vitro	—	HMGCS2 activation	—	NF-κB; autophagy	Modulated inflammatory signaling	

*Abbreviation*: *AD* Alzheimer’s disease, *MCI* mild cognitive impairment, *KD* ketogenic diet, *DHA* docosahexaenoic acid, *EPA* eicosatetraenoic acid, *NF-κB* nuclear factor kappa B, *SCFAs* short-chain fatty acids, *rmTBI* repetitive mild traumatic brain injury, *Wk*. Week, *MO* Month

The clinical studies were conducted across Asia, Europe, and North America and primarily enrolled older adults diagnosed with mild cognitive impairment (MCI) or mild-to-moderate Alzheimer’s disease (AD). Sample sizes ranged from 17 to 279 participants, with intervention durations varying between 6 weeks and 24 months. The preclinical investigations predominantly used transgenic AD mouse models, aged rodents, or cultured microglial cell systems, with experimental durations ranging from 48 h in in vitro studies to 5 months in animal models.

No restrictions were applied to the type of nutritional intervention during study selection. Following application of the predefined eligibility criteria, five principal intervention categories emerged: ketogenic dietary interventions or ketone-based strategies (*n* = 10), omega-3 fatty acids (*n* = 4), B-vitamin supplementation (*n* = 3), vitamin D supplementation (*n* = 1), and microbiota-targeted interventions (*n* = 2). One additional experimental study used a high-fat diet as a model of diet-induced neuroinflammation rather than as a therapeutic nutritional intervention.

Neuroinflammatory outcomes were assessed using a broad range of molecular, biochemical, and histopathological measures. Frequently evaluated outcomes included concentrations of TNF-α, IL-1β, IL-6, C-reactive protein (CRP), markers of microglial activation, NF-κB signaling, oxidative stress biomarkers, and indicators of gut-brain axis modulation. Considerable heterogeneity was observed in intervention protocols, biomarker panels, laboratory methodologies, and follow-up duration, precluding quantitative synthesis.

To facilitate interpretation, the findings are presented according to study design. Clinical evidence is summarized first, followed by preclinical animal studies and mechanistic in vitro investigations, before integrating the principal biological mechanisms identified across the included nutritional interventions.

### Risk of bias assessment

**Fig. 2:**
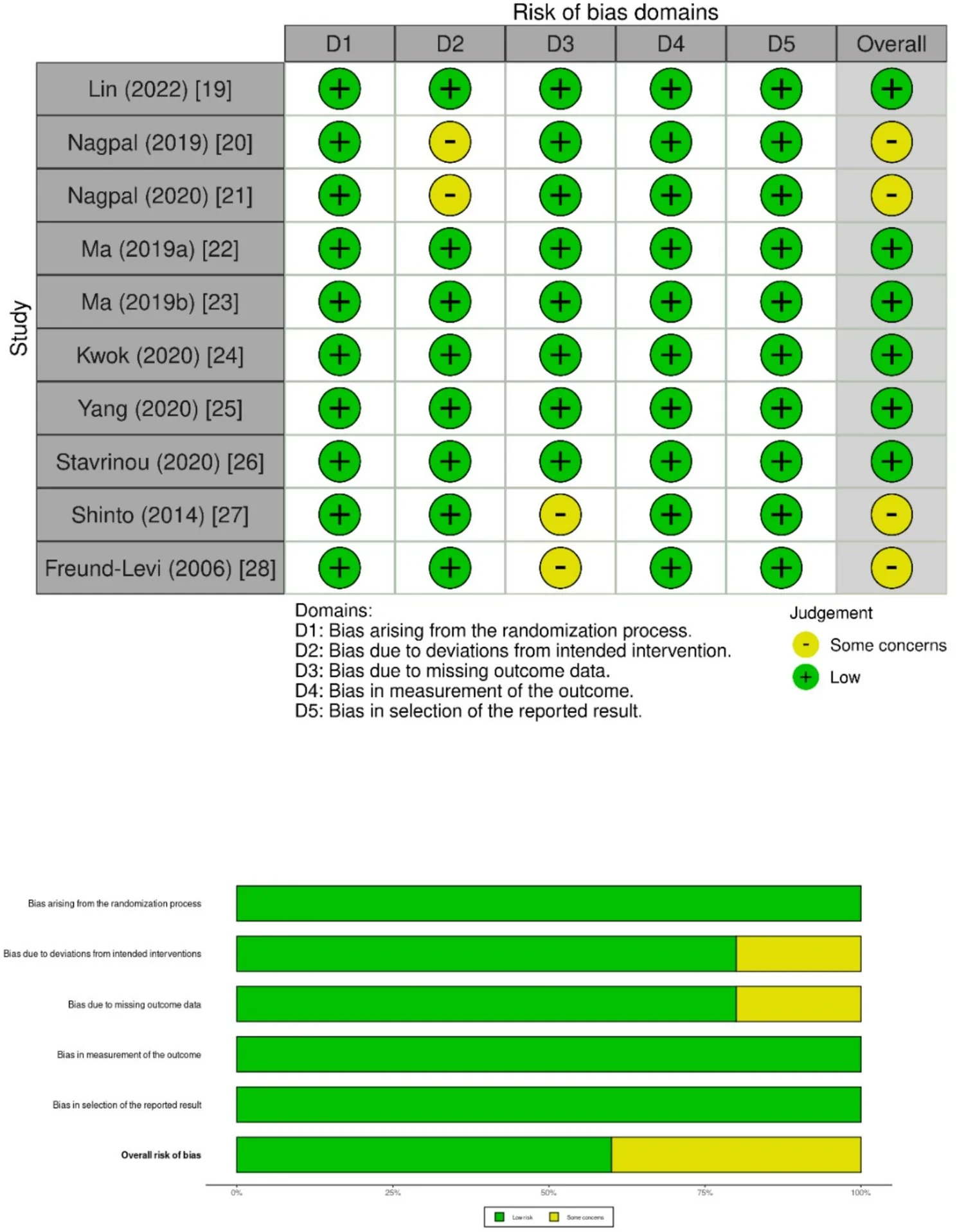
Risk of bias of randomized controlled trials (RCTs) included in the review. (**a**) showing the risk of bias of individual studies; (**b**) showing the summary of risk of bias in the RCT studies

The methodological quality of the included studies was evaluated using risk-of-bias tools appropriate for each study design. The 10 randomized controlled trials were assessed with the Cochrane Risk of Bias 2 (RoB 2) tool (Figure 2), the 10 animal studies were evaluated using the SYRCLE Risk of Bias tool, and the two mechanistic in vitro studies were appraised according to key methodological quality criteria, including experimental replication, use of appropriate controls, validity of outcome measurements, and reporting transparency. Detailed assessments are presented in Tables 3, 4 and 5.

**Table 3:** Risk of bias assessment of included clinical randomized controlled trials using the Cochrane RoB 2 Tool

Study (Ref)	Randomization Process	Deviations from Intended Interventions	Missing Outcome Data	Measurement of the Outcome	Selection of the Reported Result	Overall Risk of Bias
Lin (2022) [19]	Low	Low	Low	Low	Low	Low
Nagpal (2019) [20]	Low	Some concerns	Low	Low	Low	Some concerns
Nagpal (2020) [21]	Low	Some concerns	Low	Low	Low	Some concerns
Ma(2019a) [22]	Low	Low	Low	Low	Low	Low
Ma (2019b) [23]	Low	Low	Low	Low	Low	Low
Kwok (2020) [24]	Low	Low	Low	Low	Low	Low
Yang (2020) [25]	Low	Low	Low	Low	Low	Low
Stavrinou (2020) [26]	Low	Low	Low	Low	Low	Low
Shinto (2014) [27]	Low	Low	Some concerns	Low	Low	Some concerns
Freund-Levi (2006) [28]	Low	Low	Some concerns	Low	Low	Some concerns

*Abbreviations*: *RoB 2* Cochrane Risk of Bias 2 tool

Judgment categories: Low = low risk of bias; Some concerns = some concerns regarding bias; High = high risk of bias

**Table 4:** Risk of bias assessment of animal studies (SYRCLE Tool)

Study (Ref)	Sequence Generation	Baseline Characteristics	Allocation Concealment	Random Housing	Blinding (Caregivers)	Blinding (Outcome Assessment)	Incomplete Data	Selective Reporting	Overall Risk
Xu (2022) [29]	Unclear	Low	Unclear	Unclear	Unclear	Low	Low	Low	Some concerns
Dilimulati (2023) [30]	Unclear	Low	Unclear	Unclear	Unclear	Low	Low	Low	Some concerns
Reilly (2020) [31]	Low	Low	Unclear	Unclear	Unclear	Low	Low	Low	Some concerns
Aso (2013) [32]	Unclear	Low	Unclear	Unclear	Unclear	Low	Low	Low	Some concerns
Beckett (2013) [33]	Unclear	Low	Unclear	Unclear	Unclear	Low	Low	Low	Some concerns
Brownlow (2013) [34]	Unclear	Low	Unclear	Unclear	Unclear	Low	Low	Low	Some concerns
Hernandez (2018) [35]	Unclear	Low	Unclear	Unclear	Unclear	Low	Low	Low	Some concerns
Kashiwaya (2013) [36]	Unclear	Low	Unclear	Unclear	Unclear	Low	Low	Low	Some concerns
Ma (2018) [37]	Unclear	Low	Unclear	Unclear	Unclear	Low	Low	Low	Some concerns
Ou (2020) [38]	Unclear	Low	Unclear	Unclear	Unclear	Low	Low	Low	Some concerns

*Abbreviations*: *SYRCLE* Systematic Review Centre for Laboratory Animal Experimentation

Judgment categories: Low = low risk of bias; High = high risk of bias; Unclear = unclear risk of bias due to insufficient reporting

**Table 5:** Quality assessment of in vitro mechanistic studies

Study (Ref)	Randomization of Experiments	Blinding	Replication	Control Groups	Outcome Measurement Validity	Overall Quality
Sabokdast (2015) [39]	Not reported	Not reported	Adequate	Yes	Validated assays	Moderate
Hu (2017) [40]	Not reported	Not reported	Adequate	Yes	Validated assays	Moderate

Abbreviations: Overall quality was assessed using predefined methodological criteria, including experimental randomization, investigator blinding, replication, appropriate control groups, and validation of outcome measurement techniques. “Not reported” indicates that the study did not explicitly describe the corresponding methodological procedure

#### Clinical studies

Overall, the methodological quality of the randomized controlled trials was moderate to high. Six studies were judged to have an overall low risk of bias [19, 22–26], whereas four were rated as presenting some concerns [20, 21, 27, 28]. No study was classified as having a high overall risk of bias.

Random sequence generation was adequately described in all trials. Allocation concealment and blinding procedures were appropriately implemented in most supplementation studies, particularly those evaluating omega-3 fatty acids, B-vitamin supplementation, and vitamin D [19, 22–26]. Some concerns were identified in the ketogenic dietary crossover trials [20, 21], primarily because of the inherent challenges associated with dietary adherence and deviations from the intended intervention. Outcome data were generally complete; however, two longer-duration supplementation trials reported moderate attrition rates [27, 28], resulting in some concerns regarding incomplete outcome data. Outcome assessment relied on validated biochemical methods across all studies, and no evidence of selective outcome reporting was identified.

#### Preclinical animal studies

The methodological quality of the animal studies was considered moderate overall. Baseline characteristics were generally comparable between experimental groups, outcome data were complete, and no evidence of selective reporting was identified [29–38]. Nevertheless, several methodological domains were frequently rated as unclear because details regarding sequence generation, allocation concealment, random housing, and caregiver blinding were incompletely reported. Blinding of outcome assessment was described in several investigations, particularly those incorporating histological and molecular analyses [29, 30, 34–36, 38]. Importantly, none of the animal studies was judged to have a high overall risk of bias. Most methodological concerns reflected incomplete reporting rather than clear deficiencies in study conduct.

#### In vitro mechanistic studies

The two mechanistic in vitro studies [39, 40] were evaluated using criteria specific to experimental laboratory research rather than conventional clinical risk-of-bias instruments. Both investigations included appropriate control conditions, replicated experiments, clearly described intervention protocols, and validated molecular techniques for assessing inflammatory signaling pathways. However, neither study explicitly reported randomization of experimental conditions or investigator blinding during outcome assessment. Consequently, both studies were considered to provide moderate methodological quality, with the principal limitations related to reporting transparency rather than experimental design.

Overall, the included evidence was considered methodologically robust. The clinical trials generally demonstrated a low risk of bias, whereas the preclinical animal and mechanistic in vitro studies provided supportive biological evidence with moderate methodological confidence. Across the experimental literature, the primary limitations were attributable to incomplete methodological reporting rather than substantial threats to internal validity.

### Evidence certainty assessment

The overall certainty of evidence was evaluated using a structured narrative approach because the included studies comprised heterogeneous clinical trials, preclinical animal experiments, and mechanistic in vitro investigations. Given this substantial methodological heterogeneity, a formal GRADE assessment was not considered appropriate. Instead, evidence certainty was evaluated across four predefined domains: methodological quality (risk of bias), consistency of findings, directness of the available evidence, and translational relevance. A summary of the certainty assessment for each nutritional intervention category is presented in Table 6.

**Table 6:** Summary of overall strength of evidence across nutritional intervention categories

Nutritional intervention	Clinical studies (*n*)	Preclinical studies (*n*)	Main findings	Overall strength of evidence
Omega-3 fatty acids	4	0	Consistent association with reduced neuroinflammatory biomarkers, particularly IL-6 and TNF-α	**Moderate**
Ketogenic dietary interventions	2	8	Consistent reductions in neuroinflammatory markers in experimental models, with limited but supportive clinical evidence	**Moderate**
B-vitamin supplementation	3	0	Possible reductions in homocysteine-related inflammatory pathways, with variable biomarker responses	**Low to Moderate**
Vitamin D	1	0	Single clinical trial suggesting improvement in oxidative stress-related inflammatory processes	**Low**
Microbiota-targeted interventions	2	1	Preliminary evidence supporting modulation of gut-brain inflammatory signaling	**Low**

*Abbreviations*: *IL-6* interleukin-6, *TNF-α* tumor necrosis factor-alpha

Omega-3 fatty acids demonstrated moderate certainty of evidence, supported by multiple randomized controlled trials reporting relatively consistent reductions in neuroinflammatory biomarkers, particularly TNF-α and IL-6. Ketogenic dietary interventions were also judged to provide moderate certainty, primarily because of consistent mechanistic findings across numerous preclinical studies. Nevertheless, confidence in their clinical effectiveness remains limited because only a small number of human intervention studies met the eligibility criteria.

The certainty of evidence for B-vitamin supplementation was considered low to moderate, reflecting variability in inflammatory outcomes and substantial differences in intervention protocols across studies. Vitamin D supplementation and microbiota-targeted interventions were each supported by only a limited number of eligible studies and were therefore classified as low-certainty evidence. Consequently, conclusions regarding these interventions should be interpreted cautiously until additional well-designed randomized controlled trials become available.

Overall, the certainty of evidence differed considerably across nutritional intervention categories. Accordingly, the conclusions of this review should be interpreted in proportion to the quantity, methodological quality, consistency, and clinical applicability of the available evidence.

### Clinical evidence

Ten clinical intervention studies evaluated the effects of nutritional interventions on neuroinflammatory outcomes in individuals with Alzheimer’s disease (AD) or mild cognitive impairment (MCI). Most investigations were randomized controlled trials assessing changes in neuroinflammatory biomarkers together with cognitive and metabolic outcomes. The clinical evidence is presented according to nutritional intervention category.

#### Omega-3 fatty acids

Four randomized controlled trials investigated omega-3 fatty acid supplementation in individuals with AD or MCI [19, 26–28]. Intervention durations ranged from 6 to 12 months, with supplementation consisting primarily of docosahexaenoic acid (DHA) and eicosapentaenoic acid (EPA).

Across the included studies, omega-3 supplementation was generally associated with reductions in circulating neuroinflammatory biomarkers, particularly TNF-α and IL-6 [19, 26–28]. Several studies also reported decreases in C-reactive protein (CRP), although the magnitude of these changes varied according to baseline inflammatory status and participant characteristics [19, 26]. Improvements in oxidative stress biomarkers were observed in some trials [27], suggesting that omega-3 fatty acids may modulate interconnected inflammatory and oxidative pathways.

Although modest improvements in cognitive performance were reported in selected studies, these findings were inconsistent across trials and should therefore be interpreted cautiously. Overall, omega-3 fatty acids provided the most consistent clinical evidence for modulation of neuroinflammatory biomarkers among the nutritional interventions included in this review.

#### B-Vitamin supplementation

Three randomized controlled trials evaluated folic acid or B-vitamin supplementation in older adults with MCI [22–24]. Intervention durations ranged from 6 to 24 months.

The included studies suggested that B-vitamin supplementation may attenuate homocysteine-associated inflammatory processes, with several trials reporting reductions in neuroinflammatory biomarkers, including IL-6 and CRP [22–24]. However, the magnitude and consistency of these effects varied across studies, likely reflecting differences in baseline nutritional status, intervention protocols, treatment duration, and participant characteristics.

Overall, the available clinical evidence suggests a potential anti-inflammatory effect of B-vitamin supplementation. Nevertheless, the limited number of studies and variability in reported outcomes reduce confidence in these findings.

#### Vitamin D supplementation

One randomized controlled trial evaluated vitamin D supplementation in individuals with MCI over a 12-month intervention period [25].

The study reported improvements in oxidative stress-related neuroinflammatory biomarkers following vitamin D supplementation, suggesting a potential role in modulating neuroinflammatory processes. Nevertheless, because these findings are derived from a single clinical investigation, the available evidence remains limited. Consequently, conclusions regarding the anti-inflammatory effects of vitamin D supplementation should be interpreted cautiously until confirmed by additional randomized controlled trials.

#### Ketogenic dietary interventions

Two randomized crossover trials investigated a modified Mediterranean ketogenic diet in individuals with MCI [20, 21].

Both studies reported favorable changes in metabolic and inflammatory profiles following ketogenic dietary intervention. In addition to improvements in neuroinflammatory biomarkers, ketogenic dietary interventions were associated with alterations in gut microbiota composition, suggesting a potential interaction between ketogenic metabolism and the gut-brain axis [20, 21]. Interpretation of these findings should consider the limited number of clinical studies, relatively short intervention durations, and crossover study designs.

Overall, the available clinical evidence suggests that ketogenic dietary interventions may influence neuroinflammatory pathways in individuals with MCI. Nevertheless, the clinical evidence remains considerably smaller than the corresponding preclinical literature and should therefore be interpreted cautiously.

Because several nutritional interventions, particularly ketogenic dietary strategies, have been investigated more extensively in experimental models than in human studies, the following section summarizes the preclinical evidence supporting the biological mechanisms underlying the observed clinical findings.

### Preclinical evidence

#### Animal studies

Ten experimental animal studies investigated the effects of nutritional interventions on neuroinflammatory pathways relevant to Alzheimer’s disease (AD) and neurodegeneration [29–38]. Most studies employed transgenic AD mouse models or experimentally induced neuroinflammatory models and primarily evaluated molecular, biochemical, and histopathological markers of inflammation rather than behavioral or cognitive outcomes.

##### Ketogenic dietary interventions

Ketogenic dietary interventions represented the largest category of preclinical evidence included in this review (*n* = 8) [29, 30, 32–37]. Across these studies, ketogenic diets or ketone-based therapies consistently attenuated neuroinflammatory responses in experimental AD models. Frequently reported findings included reduced expression of pro-inflammatory cytokines, including TNF-α, IL-1β, and IL-6, suppression of NF-κB signaling, inhibition of NLRP3 inflammasome activation, reduced microglial activation, and improvements in mitochondrial bioenergetics.

Several investigations also demonstrated enhanced neuronal energy metabolism, increased expression of enzymes involved in ketone utilization, preservation of synaptic integrity, and reductions in oxidative stress. Collectively, these findings support the biological plausibility that ketogenic metabolism exerts neuroprotective effects through coordinated regulation of inflammatory and metabolic pathways. Nevertheless, because these observations were obtained predominantly from experimental animal models, they should be interpreted as mechanistic evidence that supports, rather than confirms, potential clinical efficacy.

##### High-fat diet and other experimental nutritional interventions

In contrast to the protective effects observed with ketogenic interventions, one experimental study demonstrated that chronic consumption of a high-fat diet accelerated Alzheimer’s disease pathology and exacerbated neuroinflammatory responses in APP/PS1 mice [31]. The high-fat diet increased microglial activation, enhanced inflammatory signaling, and promoted disease progression, highlighting the importance of dietary composition in regulating neuroinflammatory processes. These findings emphasize that beneficial effects cannot be attributed simply to increased dietary fat intake but appear to depend on the distinct metabolic adaptations induced by ketogenic dietary strategies.

Additional experimental studies investigated microbiota-targeted nutritional interventions [38]. These studies suggested that modulation of gut microbial composition and systemic immune signaling influenced inflammatory responses within the central nervous system. Reported effects included reductions in pro-inflammatory cytokine expression, attenuation of oxidative stress, restoration of microglial homeostasis, and improvements in gut-brain communication.

Overall, the preclinical evidence consistently demonstrated that nutritional interventions can modulate multiple biological pathways implicated in neuroinflammation. However, substantial heterogeneity in experimental models, dietary protocols, intervention duration, and outcome measures limited direct comparisons across studies and should be considered when translating these findings to clinical practice.

#### In vitro mechanistic studies

Two mechanistic in vitro studies investigated the direct effects of nutritional interventions on inflammatory signaling pathways using cultured neuronal and microglial cell models [39, 40].

Both studies demonstrated attenuation of inflammatory signaling following nutritional intervention, including suppression of NF-κB activation, reduced production of pro-inflammatory cytokines, and decreased oxidative stress-related responses. The studies also provided evidence that nutritional compounds may regulate intracellular pathways involved in energy metabolism and inflammatory signaling.

Although these mechanistic investigations provide valuable biological insights, their findings should be interpreted as complementary evidence because simplified cell culture systems cannot fully reproduce the complex cellular interactions and pathological processes occurring within the human brain.

### Mechanistic integration

Across the included studies, several biological mechanisms were consistently associated with the nutritional modulation of neuroinflammation. The most frequently reported pathways included regulation of pro-inflammatory cytokine production, attenuation of nuclear factor kappa B (NF-κB) signaling, reduction of oxidative stress, modulation of microglial activation, and improvements in mitochondrial function and cellular energy metabolism.

These mechanisms were not evaluated uniformly across all nutritional interventions. Omega-3 fatty acids were primarily associated with reductions in circulating neuroinflammatory biomarkers and enhancement of inflammation-resolution pathways in clinical studies. By comparison, ketogenic dietary interventions were investigated predominantly in preclinical models, where they consistently demonstrated inhibition of NLRP3 inflammasome activation, improved mitochondrial bioenergetics, suppression of NF-κB signaling, and modulation of immunometabolic pathways. B-vitamin supplementation was mainly associated with regulation of homocysteine-related inflammatory processes, whereas evidence for vitamin D supplementation and microbiota-targeted interventions remained limited because relatively few eligible studies were identified.

Taken together, these findings indicate that nutritional interventions may influence multiple interconnected pathways involved in neuroinflammation. Nevertheless, the apparent convergence of these mechanisms should be interpreted cautiously because individual pathways were investigated using different study designs, experimental models, intervention protocols, and outcome measures. Consequently, the available evidence supports the biological plausibility of nutritional modulation of neuroinflammatory processes rather than a single unified mechanistic framework applicable across all nutritional interventions.

## Discussion

The present systematic review focused specifically on neuroinflammatory mechanisms in Alzheimer’s disease (AD) and mild cognitive impairment (MCI), as persistent activation of innate immune pathways is increasingly recognized as a major contributor to disease onset and progression. Neuroinflammation, however, should not be viewed as an isolated pathological process. Rather, it forms part of a complex pathogenic network that interacts closely with amyloid-beta (Aβ) accumulation, tau hyperphosphorylation, synaptic dysfunction, mitochondrial dysfunction, and progressive neuronal loss. Consequently, the mechanistic findings synthesized in this review should be interpreted within the context of these interconnected pathological processes.

The review was designed primarily around AD and MCI, with clinical evidence restricted to individuals diagnosed with AD or MCI. Selected preclinical animal and in vitro models were additionally considered when they provided experimentally validated mechanistic evidence relevant to neuroinflammatory, metabolic, mitochondrial, or neuroimmune pathways implicated in AD/MCI. Accordingly, findings from these preclinical models were interpreted as supportive mechanistic evidence that helps explain biological pathways relevant to AD/MCI, rather than as direct evidence of clinical efficacy in these populations. The nutritional intervention categories summarized in this review emerged from the available eligible evidence rather than being predetermined before study selection and therefore should not be interpreted as representing the complete spectrum of nutritional strategies with potential neuroprotective properties.

### Principal clinical findings

This systematic review synthesized evidence from 22 eligible studies investigating the effects of nutritional interventions on neuroinflammatory processes relevant to AD and MCI. The clinical evidence was restricted to studies involving individuals with AD or MCI, whereas selected preclinical investigations were included to provide complementary mechanistic evidence concerning neuroinflammatory, metabolic, mitochondrial, and neuroimmune pathways relevant to AD-associated pathology. By integrating findings from randomized controlled trials with supportive preclinical and mechanistic investigations, the review provides a comprehensive overview of the current evidence while clearly distinguishing observations derived from human studies from those obtained in experimental models. Collectively, the available evidence indicates that several nutritional interventions may modulate biological pathways involved in neuroinflammation. However, the quantity, methodological quality, consistency, and certainty of evidence differed considerably across intervention categories [19–40]. Among the evaluated interventions, omega-3 fatty acids demonstrated the strongest and most consistent clinical evidence. Across the included randomized controlled trials, omega-3 supplementation was generally associated with reductions in circulating neuroinflammatory biomarkers, particularly TNF-α and IL-6, together with improvements in oxidative stress markers [19, 26–28]. Some studies also reported modest improvements in cognitive performance; however, these findings were inconsistent across trials and should be interpreted cautiously because of differences in intervention duration, dosage, participant characteristics, and outcome measures [19, 26–28]. Nevertheless, the consistency of biomarker responses suggests that omega-3 fatty acids may contribute to modulation of inflammatory processes relevant to AD and MCI.

Clinical evidence supporting ketogenic dietary interventions was more limited. Only two randomized crossover trials evaluating a modified Mediterranean ketogenic diet satisfied the eligibility criteria [20, 21]. These studies reported improvements in metabolic profiles accompanied by favorable changes in selected neuroinflammatory biomarkers and gut microbiota composition [20, 21]. Although these findings are encouraging, the small number of clinical studies, relatively short intervention periods, and crossover study designs reduce confidence in the available evidence. Consequently, the clinical findings should be interpreted independently from the substantially larger body of mechanistic evidence generated in experimental models.

Evidence supporting B-vitamin supplementation was similarly limited. Three randomized controlled trials evaluating folic acid or B-vitamin supplementation in individuals with MCI suggested potential reductions in homocysteine-associated inflammatory processes [22–24]. Nevertheless, inflammatory biomarker responses varied across studies, and the magnitude of the reported effects was inconsistent [22–24]. Differences in baseline nutritional status, intervention protocols, and treatment duration may partly explain this variability. Accordingly, although B-vitamin supplementation may influence neuroinflammatory pathways through modulation of one-carbon metabolism, the current clinical evidence remains insufficient to support definitive conclusions regarding its anti-inflammatory efficacy.

Vitamin D supplementation was represented by only a single randomized controlled trial [25]. This study reported improvements in oxidative stress-related neuroinflammatory biomarkers following supplementation, providing preliminary evidence that vitamin D may contribute to neuroimmune regulation [25]. Because these findings originate from a single clinical investigation, the certainty of evidence remains low, and additional well-designed randomized controlled trials are required before firm conclusions can be established.

Microbiota-targeted interventions were evaluated in only a small number of eligible studies [20, 21, 38]. Clinical investigations suggested that modulation of the gut microbiota may improve inflammatory signaling and metabolic pathways relevant to neurodegeneration [20, 21], whereas complementary mechanistic evidence was reported in experimental models [38]. Considerable heterogeneity in probiotic formulations, dietary protocols, participant characteristics, and outcome measures limited direct comparisons among studies. Therefore, although the gut-brain axis represents a biologically plausible therapeutic target, current clinical evidence remains preliminary.

An important finding of this review is the marked variation in evidence strength across nutritional intervention categories. Omega-3 fatty acids were supported by the largest body of consistent clinical evidence [19, 26–28], whereas vitamin D supplementation [25] and microbiota-targeted interventions [20, 21, 38] were represented by only a limited number of studies. Similarly, although ketogenic dietary or ketone-based strategies demonstrated encouraging findings, the available clinical evidence [20, 21] remains substantially smaller than the corresponding preclinical literature [29, 30, 32–37]. These differences emphasize that each nutritional intervention should be interpreted according to the quality and quantity of its individual evidence base rather than assuming equivalent efficacy across nutritional strategies.

Despite differences in intervention type and study design, several common patterns emerged across the clinical evidence. Nutritional interventions were consistently associated with modulation of neuroinflammatory biomarkers implicated in AD pathophysiology, including TNF-α, IL-1β, IL-6, and C-reactive protein, together with improvements in oxidative stress-related measures [19–28]. Nevertheless, improvements in biomarkers were not consistently accompanied by clinically meaningful improvements in cognitive function, functional status, or disease progression [19–28]. These findings indicate that biomarker modulation alone should not be interpreted as evidence of disease modification.

Overall, the available clinical evidence supports the hypothesis that selected nutritional interventions may modulate neuroinflammatory processes relevant to AD and MCI. These findings are consistent with recent systematic reviews reporting beneficial effects on inflammatory signaling and oxidative stress while concluding that current evidence remains insufficient to establish clinically meaningful disease-modifying effects because of methodological heterogeneity, limited sample sizes, and variability in intervention protocols [41]. Consequently, nutritional modulation of neuroinflammation remains biologically plausible, but its therapeutic effectiveness requires confirmation in larger, rigorously designed randomized controlled trials.

### Mechanistic insights from preclinical evidence

The preclinical evidence synthesized in this review provides important mechanistic support for the hypothesis that nutritional interventions can influence neuroinflammatory processes implicated in Alzheimer’s disease (AD). Unlike clinical studies, which predominantly assessed circulating inflammatory biomarkers and cognitive performance, animal and in vitro investigations enabled direct examination of molecular pathways involved in immune activation, oxidative stress, mitochondrial function, and neuron-glia interactions. Although these experimental findings cannot be interpreted as direct evidence of clinical efficacy, they provide valuable biological explanations for the changes observed in human intervention studies and help clarify how nutritional strategies may influence disease-related inflammatory cascades.

One of the most consistently identified mechanisms was the regulation of the nuclear factor kappa B (NF-κB) signaling pathway, a master transcription factor controlling the expression of numerous pro-inflammatory mediators, including tumor necrosis factor alpha (TNF-α), interleukin (IL)-1β, IL-6, inducible nitric oxide synthase, and cyclooxygenase-2 [42–46]. Chronic activation of NF-κB contributes to sustained microglial activation and persistent neuroinflammation, both of which accelerate neuronal dysfunction and disease progression. Across the preclinical studies evaluating ketogenic dietary or ketone-based strategies, reduced NF-κB activation was associated with lower expression of downstream inflammatory mediators and attenuation of inflammatory signaling [29, 30, 32–37]. In contrast, Reilly et al. [31] demonstrated that chronic consumption of a conventional high-fat diet accelerated amyloid pathology and enhanced neuroinflammatory responses in APP/PS1 mice, highlighting that increased dietary fat intake alone does not confer neuroprotection. Rather, the anti-inflammatory effects observed with ketogenic interventions appear to depend on the metabolic adaptations associated with nutritional ketosis, including ketone body production and altered cellular energy metabolism, rather than on fat consumption per se. These contrasting findings emphasize the importance of distinguishing ketogenic dietary therapy from non-ketogenic high-fat dietary patterns when interpreting experimental evidence.

Another prominent mechanism involved inhibition of the NLRP3 inflammasome, an intracellular signaling complex responsible for the maturation of IL-1β and IL-18 [47]. Activation of the NLRP3 inflammasome has emerged as an important contributor to chronic neuroinflammation, amyloid-associated immune activation, and progressive neuronal injury in AD [48]. Multiple experimental studies evaluating ketogenic dietary or ketone-based strategies demonstrated that increased circulating β-hydroxybutyrate concentrations were associated with suppression of NLRP3 activation and reduced production of inflammatory cytokines [29, 30, 32–37]. Beyond serving as an alternative energy substrate, β-hydroxybutyrate also functions as a signaling molecule capable of regulating immunometabolic pathways and inflammatory responses [49]. Nevertheless, these findings originate primarily from experimental models, and confirmation in larger human studies remains necessary.

Microglial regulation was another recurring observation across the included preclinical investigations. Microglia are the resident immune cells of the central nervous system and play an essential role in maintaining neural homeostasis [50]. Under pathological conditions, however, prolonged microglial activation promotes excessive release of inflammatory cytokines, reactive oxygen species, and nitric oxide, thereby contributing to synaptic dysfunction and neuronal degeneration [44, 51]. Several animal studies reported reductions in activated microglia following nutritional interventions, particularly ketogenic dietary or ketone-based strategies [29, 30, 32–37]. Some investigations further suggested a shift from pro-inflammatory toward more homeostatic microglial phenotypes, indicating restoration of immune balance within the brain. Conversely, the high-fat diet model investigated by Reilly et al. [31] was associated with greater microglial activation and exacerbation of AD pathology, further supporting the concept that dietary composition and metabolic context critically influence neuroimmune responses. Although these findings provide compelling mechanistic evidence, relatively few clinical studies have directly assessed central microglial activation using cerebrospinal fluid biomarkers or molecular neuroimaging.

Oxidative stress represented another biological process consistently influenced by nutritional interventions. Excessive production of reactive oxygen species amplifies inflammatory signaling and contributes to neuronal injury, while chronic inflammation further increases oxidative damage, creating a self-perpetuating pathological cycle [52]. Experimental studies evaluating nutritional interventions consistently demonstrated reductions in oxidative stress markers together with enhancement of endogenous antioxidant defense systems [29, 30, 32–38]. These effects were particularly evident in ketogenic dietary or ketone-based studies, while clinical evidence for vitamin D supplementation also suggested potential improvements in oxidative stress-related biomarkers [25]. Restoration of redox homeostasis may therefore constitute an important indirect mechanism through which nutritional interventions attenuate neuroinflammation.

Mitochondrial metabolism also emerged as a central component linking nutrition and neuroimmune regulation. Mitochondrial dysfunction contributes to impaired ATP production, increased oxidative stress, and activation of inflammatory signaling during aging and neurodegeneration [53]. Ketogenic dietary or ketone-based strategies consistently influenced mitochondrial bioenergetics through increased ketone utilization and altered cellular energy metabolism [29, 30, 32–37]. Several studies also reported increased expression of enzymes involved in ketone metabolism, including mitochondrial 3-hydroxy-3-methylglutaryl-CoA synthase (HMGCS2), indicating enhanced endogenous ketogenesis [37]. Improved mitochondrial efficiency was frequently accompanied by reductions in oxidative stress and inflammatory activation. By contrast, conventional high-fat feeding has been associated with worsening neuroinflammatory pathology and accelerated disease progression [31], emphasizing that the beneficial metabolic effects of ketogenic diets should not be equated with the effects of high-fat diets that do not induce nutritional ketosis.

The gut-brain axis has recently emerged as another important pathway through which nutritional interventions may influence neuroinflammation. Alterations in gut microbial composition can modify peripheral immune responses, intestinal barrier integrity, and communication between the gastrointestinal tract and the central nervous system [54, 55]. Animal studies included in this review suggested that ketogenic dietary interventions and microbiota-targeted approaches may alter microbial composition and short-chain fatty acid production [30, 37, 38]. These microbial metabolites have been associated with improved intestinal barrier function, enhanced regulatory T-cell activity, and suppression of systemic inflammatory responses that may ultimately influence neuroinflammation [56, 57]. Similar trends were reported in a limited number of clinical studies [20, 21], although differences in microbiome assessment methods, dietary protocols, and probiotic formulations reduce comparability and preclude definitive conclusions.

The available preclinical evidence further suggests that different nutritional interventions act through distinct upstream pathways while converging on common downstream inflammatory mechanisms. Omega-3 fatty acids primarily influence membrane lipid composition and the synthesis of specialized pro-resolving mediators, whereas ketogenic dietary or ketone-based strategies modify cellular energy metabolism through ketone utilization and immunometabolic regulation [45–47]. B-vitamin supplementation appears to reduce homocysteine-associated oxidative stress while supporting methylation reactions essential for neuronal function [22–24, 53]. Vitamin D exerts immunomodulatory effects through vitamin D receptor signaling within microglia and astrocytes, influencing cytokine production and inflammatory regulation [25, 54]. Despite these mechanistic differences, many interventions ultimately affect overlapping pathways involving NF-κB signaling, oxidative stress, inflammasome activation, mitochondrial dysfunction, and microglial activity, suggesting that nutritional modulation of neuroinflammation involves multiple interconnected biological processes rather than a single therapeutic target.

Overall, the experimental literature provides strong biological support for nutritional modulation of neuroinflammatory pathways relevant to AD. Nevertheless, these findings should be interpreted within the context of their experimental nature. Most mechanistic evidence originates from transgenic animal models, controlled laboratory conditions, or simplified cell culture systems that cannot fully reproduce the complexity, heterogeneity, and long disease course of sporadic late-onset AD in humans. Consequently, the mechanistic observations summarized in this review should be regarded as biologically supportive evidence that complements, rather than confirms, the clinical findings.

Although the present review focused on omega-3 fatty acids, ketogenic dietary interventions, B-vitamin supplementation, vitamin D, and microbiota-targeted approaches, other dietary bioactive compounds have also demonstrated anti-inflammatory potential in experimental models of AD. Polyphenols, particularly epigallocatechin-3-gallate (EGCG), have been reported to reduce oxidative stress, suppress microglial activation, modulate NF-κB signaling, and influence amyloid-related pathology in experimental studies [58, 59]. These findings suggest that nutritional modulation of neuroinflammation extends beyond the intervention categories represented in the current review. However, studies evaluating these compounds did not meet the predefined eligibility criteria and were therefore excluded from the qualitative synthesis. Future systematic reviews dedicated to dietary polyphenols and other emerging bioactive compounds may provide a broader understanding of their therapeutic potential in neurodegenerative disease.

### Integration of clinical and experimental findings

Integrating the clinical and preclinical evidence provides a more comprehensive understanding of the potential role of nutritional interventions in modulating neuroinflammatory processes relevant to Alzheimer’s disease (AD) and mild cognitive impairment (MCI). The clinical evidence was restricted to individuals with AD or MCI and demonstrated associations between several nutritional interventions and changes in inflammatory biomarkers. In parallel, selected experimental investigations provided mechanistic evidence explaining how these effects may occur at the cellular and molecular levels. Together, these complementary findings support the biological plausibility of nutritional modulation of neuroinflammation while emphasizing the challenges of translating mechanistic observations into clinical benefit [19–40].

Across the included randomized controlled trials, nutritional interventions were primarily associated with reductions in circulating inflammatory biomarkers, including TNF-α, IL-1β, IL-6, and C-reactive protein, together with modest improvements in oxidative stress-related measures [19–28]. Nevertheless, only a limited number of clinical studies directly evaluated central nervous system inflammation using cerebrospinal fluid biomarkers or neuroimaging techniques. Consequently, changes in peripheral inflammatory markers cannot be assumed to accurately reflect neuroinflammatory activity within the brain. By comparison, experimental animal and in vitro studies enabled direct assessment of microglial activation, astrocytic responses, inflammatory signaling pathways, mitochondrial function, and brain tissue pathology, thereby providing mechanistic evidence that is not readily obtainable in clinical investigations [29–40].

Despite considerable differences in nutritional composition and primary mechanisms of action, several interventions appeared to converge on common biological pathways involved in neuroinflammation. Omega-3 fatty acids were associated with modulation of specialized pro-resolving lipid mediators and suppression of inflammatory cytokine production [19, 26–28, 45, 46]. Ketogenic dietary or ketone-based strategies primarily influenced cellular energy metabolism, mitochondrial function, and NLRP3 inflammasome activity through ketone-mediated immunometabolic signaling [20, 21, 29, 30, 32–37, 47, 49]. In contrast, experimental evidence from a conventional high-fat diet model demonstrated accelerated amyloid pathology, increased microglial activation, and enhanced neuroinflammatory responses [31]. These opposing findings indicate that the neuroprotective effects attributed to ketogenic interventions arise from metabolic adaptations associated with nutritional ketosis rather than from increased dietary fat intake itself. B-vitamin supplementation was linked to reduced homocysteine-associated inflammatory stress [22–24, 53], whereas vitamin D appeared to regulate innate immune responses through vitamin D receptor-mediated signaling [25, 54]. Although these interventions act through distinct upstream mechanisms, many ultimately influenced shared downstream processes involving NF-κB activation, oxidative stress, microglial activity, and neuroimmune regulation [42–49, 51–56, 60].

These mechanistic similarities should be interpreted cautiously. The included studies differed substantially in study design, intervention protocols, treatment duration, experimental models, biomarker selection, and methodological quality. Moreover, many molecular pathways identified in experimental studies were not consistently evaluated in clinical trials, limiting direct comparison between mechanistic and clinical findings. Therefore, although the available evidence suggests convergence across several inflammatory pathways, it does not indicate that all nutritional interventions operate through identical mechanisms or produce equivalent therapeutic effects in humans.

Another important observation was the unequal distribution of evidence across intervention categories. Omega-3 fatty acids were supported by multiple randomized controlled trials involving individuals with AD or MCI [19, 26–28], whereas ketogenic dietary or ketone-based strategies were supported by a smaller clinical evidence base and a substantially larger body of preclinical mechanistic evidence [20, 21, 29, 30, 32–37]. Conversely, vitamin D supplementation, B-vitamin supplementation, and microbiota-targeted interventions were represented by relatively few eligible studies [20–25, 38]. Consequently, the certainty and consistency of evidence varied considerably among nutritional strategies and should be interpreted according to both the quantity and methodological quality of the available evidence.

**Fig. 3:**
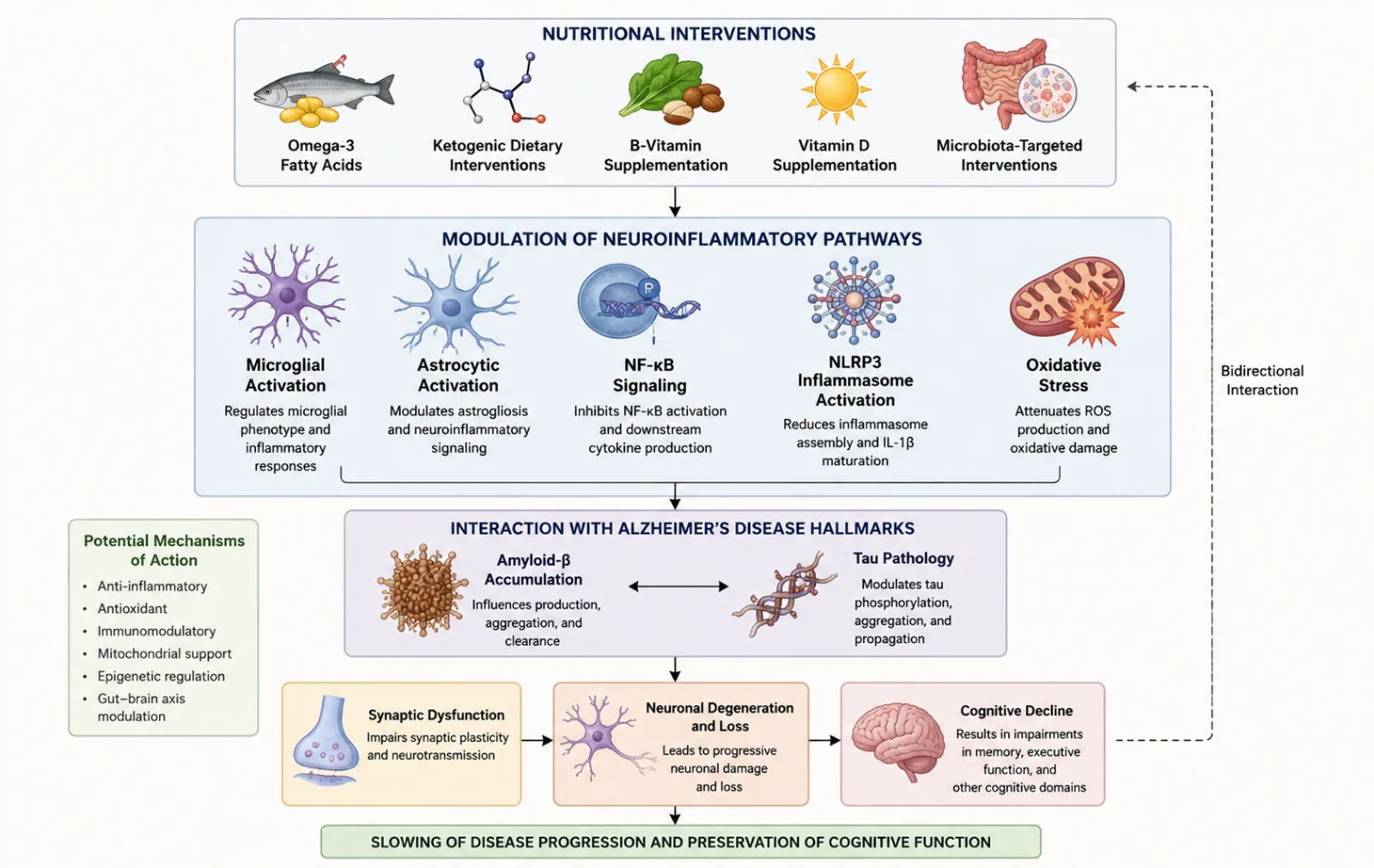
Proposed mechanistic framework linking nutritional interventions, neuroinflammation, and Alzheimer’s disease pathology

Collectively, the integrated evidence indicates that nutritional interventions may modulate interconnected neuroinflammatory pathways implicated in AD pathogenesis. However, recent systematic reviews similarly conclude that, despite encouraging effects on inflammatory biomarkers and mechanistic pathways, current clinical evidence remains insufficient to establish definitive disease-modifying efficacy because of methodological heterogeneity, limited sample sizes, variability in intervention protocols, and inconsistent outcome assessment [41, 61]. The present findings are consistent with these broader evidence syntheses and further emphasize the need for adequately powered, multicenter randomized controlled trials incorporating standardized neuroinflammatory biomarkers together with clinically meaningful cognitive and functional outcomes. A conceptual summary of the proposed interactions among nutritional interventions, neuroinflammatory mechanisms, and the pathological cascade of Alzheimer’s disease is presented in Fig. 3.

### Implications for clinical practice

The findings of this systematic review suggest that nutritional interventions may serve as complementary strategies for modulating neuroinflammatory processes associated with Alzheimer’s disease (AD) and mild cognitive impairment (MCI). Nevertheless, the current evidence does not support their use as disease-modifying therapies or as substitutes for established pharmacological and multidisciplinary management. Rather, nutritional interventions should be considered components of comprehensive care aimed at promoting overall brain health alongside optimization of cardiovascular risk factors, regular physical activity, cognitive stimulation, and individualized nutritional assessment [42, 43].

From a clinical perspective, evaluation of nutritional status may represent an important aspect of routine care for individuals with AD or MCI. Identification and correction of nutritional deficiencies, including inadequate vitamin D status or elevated homocysteine concentrations related to insufficient B-vitamin intake, may improve metabolic health while potentially influencing inflammatory pathways implicated in neurodegeneration [22–25, 54]. Similarly, maintaining adequate dietary intake of omega-3 fatty acids may support healthy brain aging, although the current evidence remains insufficient to recommend omega-3 supplementation specifically for the prevention or treatment of AD or MCI [19, 26–28].

The findings also emphasize the importance of incorporating objective biomarkers into future clinical practice and research. Standardized assessment of inflammatory cytokines, oxidative stress markers, cerebrospinal fluid biomarkers, and advanced neuroimaging techniques may improve understanding of whether nutritional interventions influence central nervous system inflammation in addition to peripheral inflammatory responses. Greater standardization of biomarker assessment would also facilitate comparisons across studies and strengthen the interpretation of treatment effects [42, 44, 55].

An additional implication is the potential role of precision nutrition in the management of neurodegenerative disorders. Clinical responses to nutritional interventions are likely influenced by baseline nutritional status, metabolic health, genetic susceptibility, gut microbiota composition, comorbidities, and disease stage. Consequently, individualized nutritional strategies may prove more effective than uniform dietary recommendations. However, these personalized approaches require validation in adequately powered randomized controlled trials before they can be incorporated into routine clinical practice.

Overall, the available evidence supports the integration of nutritional assessment and evidence-based dietary counseling into comprehensive management strategies for individuals with AD and MCI. Nevertheless, recommendations regarding specific nutritional interventions should remain proportional to the strength and certainty of the available evidence, recognizing that further high-quality clinical trials are required before targeted nutritional therapies can be routinely recommended for neuroinflammatory modulation.

### Strengths and limitations

This systematic review has several important strengths. First, it provides a comprehensive synthesis of both clinical and preclinical evidence examining the effects of nutritional interventions on neuroinflammatory mechanisms relevant to Alzheimer’s disease (AD) and mild cognitive impairment (MCI). By integrating findings from randomized controlled trials, experimental animal studies, and mechanistic in vitro investigations, the review offers a translational perspective while clearly distinguishing clinical evidence from mechanistic observations. This integrated approach improves understanding of the biological rationale underlying nutritional modulation of neuroinflammation and its potential clinical relevance.

Second, the review was conducted according to the PRISMA 2020 guidelines and followed a predefined protocol registered in PROSPERO. A comprehensive search strategy across multiple electronic databases, supplemented by manual reference screening, citation tracking, and grey literature searches, minimized the likelihood of missing relevant studies. Furthermore, methodological quality was evaluated using validated, study design-specific risk-of-bias tools, including the Cochrane Risk of Bias 2 tool for randomized controlled trials and the SYRCLE Risk of Bias tool for animal studies, thereby enhancing the methodological rigor and transparency of the review.

Another strength is the structured evaluation of evidence certainty. Because the included studies encompassed heterogeneous clinical, animal, and mechanistic investigations, a formal GRADE assessment was not appropriate. Instead, evidence certainty was assessed using predefined domains, including methodological quality, consistency of findings, directness of evidence, and translational relevance. This approach provides a balanced interpretation of the available evidence while acknowledging important differences among nutritional intervention categories.

Several limitations should also be considered. Considerable methodological heterogeneity existed across the included studies with respect to intervention type, dosage, formulation, treatment duration, participant characteristics, experimental models, biomarker selection, and outcome assessment. This heterogeneity precluded quantitative meta-analysis and limited direct comparisons across studies. Moreover, most clinical trials enrolled relatively small samples and had short follow-up periods, reducing the ability to evaluate long-term effects on cognitive decline, disease progression, or sustained neuroinflammatory modulation.

Interpretation of the mechanistic evidence also requires caution. Although animal and in vitro studies provided valuable insights into inflammatory signaling pathways, mitochondrial function, and neuron-glia interactions, these experimental models cannot fully replicate the complexity, heterogeneity, and multifactorial nature of sporadic late-onset AD in humans. Consequently, mechanistic findings should be regarded as supportive evidence that strengthens biological plausibility rather than direct confirmation of clinical efficacy.

Another limitation relates to the unequal distribution of evidence across nutritional intervention categories. Omega-3 fatty acids were supported by the largest body of clinical evidence, whereas ketogenic dietary interventions were represented predominantly by preclinical investigations. In contrast, vitamin D supplementation, B-vitamin supplementation, and microbiota-targeted interventions were supported by relatively few eligible studies, resulting in lower certainty of evidence for these approaches. Therefore, conclusions regarding individual nutritional interventions should be interpreted according to the quantity, methodological quality, consistency, and clinical applicability of the available evidence.

Potential publication bias should also be acknowledged because studies reporting positive findings are generally more likely to be published than those with neutral or negative results. In addition, inconsistent reporting of baseline nutritional status, dietary intake, treatment adherence, biomarker methodology, and participant characteristics limited assessment of important effect modifiers and reduced comparability across studies. Variability in biomarker panels and laboratory methods further complicated interpretation of treatment effects.

Overall, despite these limitations, this systematic review provides a rigorous and comprehensive synthesis of the current evidence regarding nutritional modulation of neuroinflammation in AD and MCI. The findings identify important knowledge gaps and emphasize the need for larger, multicenter randomized controlled trials employing standardized intervention protocols, harmonized neuroinflammatory biomarkers, and clinically meaningful outcomes to strengthen the evidence base and improve translation into clinical practice.

### Future research

Future research should prioritize large, multicenter randomized controlled trials with adequate statistical power and longer follow-up periods to determine whether improvements in neuroinflammatory biomarkers translate into clinically meaningful benefits, including slower cognitive decline, preservation of functional capacity, and delayed disease progression. Standardized intervention protocols, well-defined participant populations, and harmonized outcome measures will be essential to improve comparability across studies and strengthen the overall quality of evidence.

A major research priority is the standardization of neuroinflammatory biomarker assessment. Future clinical trials should incorporate validated panels of inflammatory cytokines, oxidative stress markers, and neuroimmune biomarkers together with more direct measures of central nervous system inflammation, including cerebrospinal fluid analysis and advanced neuroimaging techniques, such as positron emission tomography (PET) imaging of microglial activation. Integrating peripheral and central biomarkers may clarify whether systemic anti-inflammatory effects accurately reflect changes within the brain and improve understanding of the biological mechanisms underlying nutritional interventions.

Future investigations should also examine the interaction between neuroinflammation and the core pathological processes of Alzheimer’s disease, including amyloid-beta (Aβ) accumulation, tau hyperphosphorylation, synaptic dysfunction, mitochondrial dysfunction, and neuronal degeneration. Simultaneous evaluation of these interconnected mechanisms may provide a more comprehensive understanding of disease pathophysiology and help determine whether nutritional interventions influence multiple pathological pathways rather than isolated inflammatory processes.

The development of precision nutrition approaches represents another important research direction. Individual responses to nutritional interventions are likely influenced by baseline nutritional status, metabolic health, genetic susceptibility, gut microbiota composition, lifestyle factors, and disease stage. Future studies should therefore incorporate comprehensive clinical, metabolic, genomic, and microbiome profiling to identify patient subgroups most likely to benefit from targeted nutritional strategies. Such personalized approaches have the potential to improve treatment efficacy but require prospective validation before clinical implementation.

Additional high-quality randomized controlled trials are particularly needed for nutritional interventions currently supported by limited clinical evidence, including B-vitamin supplementation, vitamin D, and microbiota-targeted therapies. Well-designed comparative studies evaluating different nutritional interventions, alone or in combination, may further clarify their relative effectiveness and potential synergistic effects on neuroinflammatory pathways.

Future studies should also place greater emphasis on clinically meaningful outcomes in addition to biomarker modulation. Although changes in inflammatory biomarkers provide valuable mechanistic information, their relationship with cognitive performance, functional independence, quality of life, and disease progression remains incompletely understood. Establishing these associations is essential for determining the clinical relevance of nutritional modulation of neuroinflammation.

As higher-quality and more homogeneous evidence becomes available, future systematic reviews should incorporate quantitative meta-analyses and formal certainty assessments to provide more robust estimates of treatment effects. In addition, emerging dietary bioactive compounds, including polyphenols such as epigallocatechin-3-gallate (EGCG), curcumin, and resveratrol, warrant dedicated systematic evaluation as the clinical evidence continues to expand. Such investigations may broaden current understanding of nutritional strategies capable of targeting neuroinflammatory pathways in Alzheimer’s disease and mild cognitive impairment.

Overall, advancing this field will require closer integration of clinical, mechanistic, and translational research. Collaborative multidisciplinary studies combining nutritional science, neuroscience, immunology, metabolomics, microbiome research, and advanced biomarker technologies will be critical for establishing whether nutritional interventions can produce sustained neurobiological and clinically meaningful benefits in individuals with Alzheimer’s disease and mild cognitive impairment.

## Conclusions

This systematic review synthesized the available clinical and preclinical evidence evaluating the effects of nutritional interventions on neuroinflammatory mechanisms relevant to Alzheimer’s disease (AD) and mild cognitive impairment (MCI). The findings indicate that several nutritional strategies, including omega-3 fatty acids, ketogenic dietary interventions, B-vitamin supplementation, vitamin D, and microbiota-targeted approaches, may influence biological pathways implicated in neuroinflammation. Across the included studies, these interventions were associated with modulation of inflammatory cytokines, microglial activity, oxidative stress, mitochondrial function, and gut-brain signaling, providing mechanistic support for a potential role in neurodegenerative processes.

The certainty of evidence differed substantially among intervention categories. Omega-3 fatty acids demonstrated the most consistent clinical evidence for modulation of neuroinflammatory biomarkers, whereas evidence supporting ketogenic dietary interventions was derived predominantly from experimental studies. By comparison, B-vitamin supplementation, vitamin D, and microbiota-targeted interventions were supported by relatively few eligible clinical studies, resulting in lower certainty of evidence.

Overall, the integrated clinical and experimental findings support the biological plausibility that nutritional interventions may modulate interconnected neuroinflammatory pathways involved in AD pathogenesis. However, the current clinical evidence remains insufficient to establish definitive disease-modifying efficacy or to support routine therapeutic recommendations. Future adequately powered, multicenter randomized controlled trials incorporating standardized neuroinflammatory biomarkers together with clinically meaningful cognitive and functional outcomes are required to determine whether the observed mechanistic effects translate into sustained clinical benefit.

## Supplementary Information

Supplementary Material 1.

Supplementary Material 2: File S1: PRISMA (Preferred Reporting Items for Systematic Reviews and Meta-Analyses) guidelines, File S2: Search strategy in different database.

## Data Availability

All the included studies are in article.
