# Supplementary material for: Nutritional modulation of neuroinflammation in Alzheimer’s disease and mild cognitive impairment: a systematic review of clinical and preclinical evidence
Source: BMC Geriatr. 2026 Aug 25;26:1100. doi: 10.1186/s12877-026-08126-x (PMC13520152; doi:10.1186/s12877-026-08126-x)
Supplement: Supplementary file 1 [file 12877_2026_8126_MOESM1_ESM.docx]

**Supplementary Table S1. Complete Electronic Database Search Strategies**

| **Database** | **Complete Search String** |
| --- | --- |
| **PubMed/MEDLINE** | (("Neuroinflammation"[Mesh] OR neuroinflamm* OR "central nervous system inflammation" OR "CNS inflammation" OR microglia* OR "microglial activation" OR astrocyt* OR "astrocyte activation" OR "pro-inflammatory cytokines" OR "tumor necrosis factor-alpha" OR TNF-alpha OR IL-1beta OR IL-6 OR "nuclear factor kappa B" OR NF-kB) AND ("Nutrients"[Mesh] OR nutrient* OR diet* OR "dietary pattern*" OR "nutrition therapy"[Mesh] OR "fatty acids, omega-3"[Mesh] OR omega-3 OR omega 3 OR DHA OR EPA OR "polyunsaturated fatty acids" OR PUFA* OR "vitamin D" OR cholecalciferol OR "vitamin E" OR tocopherol* OR antioxidant* OR polyphenol* OR flavonoid* OR curcumin OR resveratrol OR "ketogenic diet" OR probiotic* OR prebiotic* OR "gut microbiota" OR fiber OR "short-chain fatty acids") AND ("Neurodegenerative Diseases"[Mesh] OR "Alzheimer Disease"[Mesh] OR "Parkinson Disease"[Mesh] OR "Multiple Sclerosis"[Mesh] OR "Traumatic Brain Injury"[Mesh] OR stroke OR dementia OR "cognitive decline")) |
| **Embase** | ('neuroinflammation'/exp OR neuroinflamm*:ti,ab OR 'central nervous system inflammation':ti,ab OR 'microglia activation'/exp OR microglia*:ti,ab OR astrocyte*:ti,ab OR 'nf kappa b'/exp OR 'tumor necrosis factor alpha'/exp OR 'interleukin 1 beta'/exp OR 'interleukin 6'/exp) AND ('nutrient'/exp OR nutrient*:ti,ab OR diet*:ti,ab OR 'omega 3 fatty acid'/exp OR DHA:ti,ab OR EPA:ti,ab OR 'vitamin d'/exp OR 'vitamin e'/exp OR 'polyphenol'/exp OR curcumin:ti,ab OR resveratrol:ti,ab OR 'ketogenic diet'/exp OR probiotic*:ti,ab OR 'gut microbiota'/exp OR fiber:ti,ab) AND ('neurodegenerative disease'/exp OR 'alzheimer disease'/exp OR 'parkinson disease'/exp OR 'multiple sclerosis'/exp OR 'brain injury'/exp OR stroke:ti,ab OR dementia:ti,ab) |
| **Scopus** | TITLE-ABS-KEY (neuroinflamm* OR "central nervous system inflammation" OR "CNS inflammation" OR microglia* OR "microglial activation" OR astrocyt* OR "NF-kB" OR "tumor necrosis factor" OR "interleukin-1 beta" OR "interleukin-6") AND TITLE-ABS-KEY (nutrient* OR diet* OR "omega-3" OR DHA OR EPA OR "polyunsaturated fatty acids" OR "vitamin D" OR "vitamin E" OR antioxidant* OR polyphenol* OR curcumin OR resveratrol OR "ketogenic diet" OR probiotic* OR "gut microbiota" OR fiber OR "short-chain fatty acids") AND TITLE-ABS-KEY ("Alzheimer*" OR "Parkinson*" OR "multiple sclerosis" OR stroke OR dementia OR "traumatic brain injury") |
| **Web of Science Core Collection** | TS=(neuroinflamm* OR "central nervous system inflammation" OR "CNS inflammation" OR microglia* OR astrocyt* OR "NF-kB" OR "tumor necrosis factor" OR "interleukin-1 beta" OR "interleukin-6") AND TS=(nutrient* OR diet* OR "omega-3" OR DHA OR EPA OR "polyunsaturated fatty acids" OR "vitamin D" OR "vitamin E" OR antioxidant* OR polyphenol* OR curcumin OR resveratrol OR "ketogenic diet" OR probiotic* OR "gut microbiota" OR fiber OR "short-chain fatty acids") AND TS=("Alzheimer*" OR "Parkinson*" OR "multiple sclerosis" OR stroke OR dementia OR "traumatic brain injury") |
